# Seed dressing with M451 promotes seedling growth in wheat and reduces root phytopathogenic fungi without affecting endophytes

**DOI:** 10.3389/fpls.2023.1176553

**Published:** 2023-05-17

**Authors:** K. Kardava, V. Tetz, M. Vecherkovskaya, G. Tetz

**Affiliations:** Department of Systems Biology, Human Microbiology Institute, New York, NY, United States

**Keywords:** *Triticum aestivum*, fungicide, endophytes, pathogen, M451, pathogen resistance

## Abstract

Fungal plant infections result in substantial losses to the agricultural sector. A range of fungicide seed dressings are available to control seed-borne fungal diseases; however, they lack sufficient efficacy because of intrinsic tolerance and acquired resistance. Moreover, many fungicide seed dressings can also penetrate plants, negatively affecting plant growth owing to their toxic effects on endophytes, as well as contributing to the spread of antibiotic resistance. Here, we evaluated the efficacy of M451, a member of a new class of antimicrobial agents that are not relevant to human healthcare. As a seed dressing for wheat seeds, M451 exhibited significant antifungal activity against one of the most devastating plant fungal pathogens, *Fusarium* spp. Furthermore, M451 was more active than the commercially used fungicide Maxim XL against both seed-borne and soil-borne *F. oxysporum* infection. Importantly, and unlike other antifungals, M451 seed dressing did not inhibit any of the major characteristics of wheat grains and seedlings, such as germination percentage, germination time, grain vigor, shoot- and root weight and length, but rather improved some of these parameters. The results also demonstrated that M451 had no negative impacts on endophytes and did not accumulate in grains. Thus, M451 may have potential applications as an antifungal agent in wheat cultivation.

## 1 Introduction

Fungal wheat diseases, cause substantial losses in food production, being responsible for over 15% of global losses in wheat production (Kaur, 2016; Zaller et al., 2016; Simón et al., 2021). In wheat cultivation seed-borne fungal diseases are responsible for grain death, poor crop establishment, and reduced germination rates, while soil-borne root fungal diseases also cause massive destruction and crop loss, playing a vital role in reducing the profitability, quality, and quantity of production (Rajput et al., 2005). Therefore, fungicidal seed dressings are widely used to protect crop grains from fungal diseases. Seed application of antifungal compounds has unique benefits over other methods used to treat fungal plant infections, and it can be used to prevent and overcome those diseases that cannot be controlled after planting.

However, existing antifungal seed dressing agents show limited effectiveness in reducing fungal diseases in crops. This is partly because seed and root pathogenic fungi belong to diverse genera, and only a few fungicides have broad-spectrum activities against a variety of fungal pathogens, being able to lessen the severity of the infection (Fan et al., 2017; McGee, 1995; Tunali et al., 2008; Coker et al., 2010). Another relevant issue is the emergence of resistance to available fungicides, which—along with high intrinsic antifungal resistance—dramatically reduces the efficacy of available therapeutic options (Conner et al., 1996; Janmajay et al., 2011; Thind, 2012; Lucas et al., 2015). Therefore, several commercially available agricultural seed dressing fungicides contain two or more antifungal agents that target different fungal pathogens (e.g., Maxim XL contains fludioxonil and mefenoxam). However, even these combined products have limited efficacy. Moreover, they show increased toxicity in plants, resulting in lower seed germination rates and decreases in shoot and root lengths and relative water content (Kookana et al., 1998; Komárek et al., 2010; Sykes et al., 2018; Yang et al., 2019; Wen et al., 2022; Serra et al., 2015; Singh and Sahota, 2018). In addition to having a direct toxic effect on plant seeds, many agricultural fungicides inhibit plant endophytes, which are important contributors to plant growth (Baibakova et al., 2019). Indeed, many plant endophytes that belong to Aspergillus spp., Penicillium spp., and Staphylococcus spp. are sensitive to fungicide seed dressings and their loss disrupts mutualist endophyte-host interactions negatively affecting different plant characteristics (Javed et al., 2019; Błaszczyk et al., 2021).

Another concern related to many existing agricultural fungicides is that they have a chemical structure similar to that of fungicides used in human healthcare, including derivatives of azoles and polyene antibiotics (Takahashi et al., 2020). These environmental fungicides can become potential sources of resistance for invasive human mycoses owing to cross-resistance with medical antifungal agents (Takahashi et al., 2020; Bastos et al., 2021). This can happen through fungicide-exposed soil driving the selection of acquired fungal-resistant clones and/or the accumulation of these environmental fungicides inside plants, which are then consumed by humans and other animals (Bastos et al., 2021; Li et al., 2022; Elfikrie et al., 2020). As a result, there is a significant unmet need for a broad-spectrum antifungal seed dressing agent that is active against resistant fungal species, does not penetrate plants, is not toxic to endophytes, and is not a derivative of those used for the treatment of human diseases (Brauer et al., 2019).

Recently, we described the high fungicidal activity of M451, a broad-spectrum antifungal agent. M451 is a combinatorial product of TGV-28 [poly-(N-carboxamido-1,6-diaminohexane), particularly N’-aminated], which is a derivative of the antimicrobial agent Mul-1867, and NaH_2_PO_4_ (0.01%). M451 has been shown to exhibit fungicidal activity against different strains of *Ascomycota*, *Oomycota*, and *Basidiomycota* (Tetz and Tetz, 2015; Tetz et al., 2017; Tetz et al., 2023). In this study, we assessed the efficacy of M451 as a seed dressing against seed- and root-borne fungal infection in wheat. We also evaluated its effects on endosymbionts and its extent of penetration and absorption into wheat plants.

## 2 Materials and methods

### 2.1 Seed dressing preparation

Triticale grains (*Triticum aestivum* L. emend. Fiori et Paol) were obtained from local commercial suppliers. Wheat grains with known seed-borne *Fusarium* infections were obtained from the Human Microbiology Institute (New York, NY, USA). Potato dextrose agar (PDA; Thermo Fisher Scientific Inc., Waltham, MA, USA) was used as a growth medium for seed germination.

**Figure 1:**
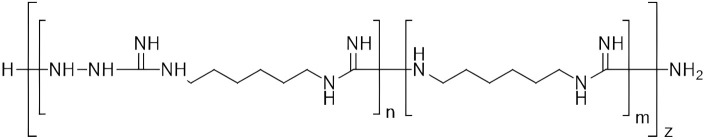
Chemical structure of TGV-28 [poly-(N-carboxamido-1,6-diaminohexane), particularly N’-aminated].

Grains in the control group were sterilized according to Htwe et al. (2011), with minor modifications. Briefly, uniformly sized grains were washed in a solution of 4.5% (w/v) NaOCl:water (1:1 ratio) for 20 min. For the experimental groups, uniformly sized grains were treated with different concentrations of three different solutions: 1) 0.1% and 0.05% M451 (a water-based solution of TGV-28 [**Figure 1**]; Human Microbiology Institute) in combination with 0.01% NaH_2_PO_4_ (Sigma Aldrich, St. Louis, MO, USA) for 30 min, 2) 0.01% NaH_2_PO_4_ for 30 min (Sigma Aldrich), and 3) Maxim XL v/v1:10 dilution in water for 12 h according to the manufacturer’s instructions (Syngenta Canada Inc., Guelph, ON, Canada) (Costa et al., 2019; Pfeiffer et al., 2021). Subsequently, the grains from control and M451-treated groups were washed three times with autoclaved distilled water and then air-dried on filter paper. The grains treated with Maxim XL were left unwashed.

### 2.2 Analysis of growth characteristics

Grains were treated with different concentrations of test compounds, as described above, or the control solution (4.5% (w/v) NaOCl:water (1:1 ratio), and the number of grains that sprouted and germinated were counted daily for up to 7 days. Sprouted grains were defined as those that produced at least one noticeable plumule or radicle (Rahman et al., 2001). The grains were considered to have germinated when at least 2 mm of radicle emerged from the grain coat. On the seventh day of germination, the following parameters were recorded: water uptake percentage, germination percentage, germination index, grain vigor, mean germination time, and biomass (Tsegay and Gebreslassie, 2014). Seedling height, shoot length, and root length of three randomly chosen seedlings were measured in each treatment and the control groups (Zhang et al., 2014).

### 2.3 Measurement of water uptake percentage

Prior to sterilizing the grains, the dry weight of each selected grain was measured using a scale (MKE-5, Arlyn Series, Arlyn Scales, East Rockaway, NY, USA). Thereafter, the grains were treated with M451 (0.1% and 0.05%), NaH_2_PO_4_ (0.01%), or Maxim XL, as described in 2.1. The grains were then transferred to Petri dishes (n =? per dish) lined with Whatman No. 1 filter paper, and 10 ml of sterile distilled water was added to each dish. The grains were incubated for 24 h at the room temperature. The fresh weight of the grains in each treatment group was recorded to determine the water uptake (Gairola et al., 2011) using the equation:


$$\text{Water uptake }\left(\%\right)=\frac{\text{seed fresh weight}-\text{seed dry weight}}{\text{seed fresh weight}}\;\times \;100$$

### 2.4 Measurement of the germination percentage

Germination percentage (GP) was calculated based on the total number of grains in each group that germinated using the equation:


$$\text{GP}\;\left(\%\right)=\frac{\text{number of germinat}ed\text{ seeds}}{\text{total number of seeds sown}}\;\times \;100$$

### 2.5 Measurement of the germination index

The germination index (GI) was calculated as the sum of germinated grains on a given day (Gt) divided by the number of germination days (Dt) using the following equation:


$$\text{GI}=\sum^{}\frac{\text{Gt}}{\text{Dt}}$$

### 2.6 Measurement of the mean germination time

The mean germination time (MGT) was determined as the average amount of time needed for a grain to initiate and terminate the germination process (Ellis and Roberts, 1981). A grain was considered to have germinated once its radicle had reached 2 mm in length (Montaña et al., 2014). MGT was calculated using the following equation:


$$\text{MGT}=\;\frac{\sum^{}\text{Dn}}{\sum^{}\text{n}}$$

where n is the number of germinated grains that germinated on day D, and D is the number of days counted from the beginning of germination.

### 2.7 Measurement of grain vigor

Grain vigor was determined to evaluate the activity level and performance of a grain during germination using a previously described formula (Abdul-Baki and Anderson, 1973) as follows:


$$Grain\text{ vigor}=\frac{l\text{ength of hypocotyl}+\text{leng}t\text{h of radical}}{100}\times \text{GP}$$

where GP is the germination percentage.

### 2.8 Measurement of dry weight

The dry weight of seedlings before and after treatment was determined by drying freshly collected seedling samples at 80°C for 24 h. Dry weight was measured in mg using a scale (MKE-5, Arlyn Series) (Carpýcý et al., 2009).

### 2.9 Measurement of shoot and root length

After 7 d of germination in a plastic pot, the length of normal seedlings and the corresponding shoot and root length were recorded in cm (Oukhouia et al., 2017).

### 2.10 Isolation of endophytic bacteria and fungi

Grains sterilized with 4.5% NaOCl:water (1:1) or treated with 0.1% M451 were cut in half and plated on PDA medium, and then incubated for 120 h at 25°C. Fungi were identified based on the morphological parameters of the colonies, such as colony shape, pigmentation, the presence of septa, and the shape of the conidia. For morphological identification, fungal fragments were placed on a glass slide, stained with methylene blue, and observed under light microscopy (Axiostar plus, Carl Zeiss, Germany; objective lens: A-Plan 100х/1.25). Bacteria were identified based on proteome analysis using matrix-assisted laser desorption/ionization-time of flight (MALDI-TOF) mass spectrometry (MS) with sample preparation on AnchorChip microtiter plates (Bruker Corporation, Billerica, MA, USA). The bacteria were identified using the MALDI Biotyper Database (Bruker Taxonomy Tree, Bruker Corporation).

### 2.11 Germination of soil-borne *Fusarium* grains

*Fusarium oxysporum* conidia, confirmed by molecular identification, were harvested from 5-d-old cultures grown on PDA medium, mixed with sterile water, and then mixed with soil at a spore density of 2 × 10^5^ g^–1^ dry weight soil, as previously described (Oukhouia et al., 2017). Grains in the control and fungicide-treated groups were seeded in a single row (length, 20 cm; 10 grains per row; depth, 2 cm) and completely covered with soil. A total of seven rows were planted per container, spaced 5 cm apart. After planting, the containers were placed in growth chambers at a constant temperature with a 12-h light cycle.

### 2.12 Analysis of M451 accumulation in plants

For the quantitative analysis of M451 accumulation in plants, we used untreated seedlings as a negative control, seedlings from seeds treated with 0.1% sulfamethoxazole (Sigma Aldrich) as a positive seed dressing control, and seedlings from seeds treated with 0.1% M451 seed dressing as the test group. The samples were freeze-dried for 24 h for this analysis (Jones-Lepp et al., 2010). The extraction and cleanup of the samples were performed as previously described by Pan et al. (2014). The extracted samples were examined using ultra-high-performance liquid chromatography coupled with quadrupole-linear ion-trap tandem MS (UPLC-MS/MS; LC-20 Prominence HPLC system coupled with a LCMS-8050 triple quadrupole mass spectrometer; Shimadzu, Tokyo, Japan) as described in Gros et al. (2013). The chromatographic and MS conditions for the analysis of M451 accumulation are described in **Supplementary Table 1**.

### 2.13 Biochemical composition of wheat seedlings

For the phytochemical profiling of wheat seedlings, a 100 g sample was collected from each group in triplicate. Dry matter was measured gravimetrically (AOAC 931.04), Bertrand’s method was used to determine the sugar content (Badger, 2002), and ascorbic acid levels were measured spectrophotometrically (SmartSpec Plus, Bio-Rad Laboratories, Hercules, CA, USA) (Salachna et al., 2018). Carotenoid and chlorophyll content was evaluated at different wavelengths according to Schüler et al. (2020) (**Supplementary Table 2**).

### 2.14 Statistical analysis

For all data, appropriate descriptive statistics are presented in the supplementary tables. Between-group differences were evaluated using one-way analysis of variance (ANOVA) in the GraphPad Prism software (version 9.3.1). Differences were considered statistically significant at *P*<0.05. All experiments were performed in triplicate and the results are presented as the mean ± standard deviation (SD).

## 3 Results

### 3.1 Effect of M451 seed dressing on seed-borne pathogens

**Figure 2:**
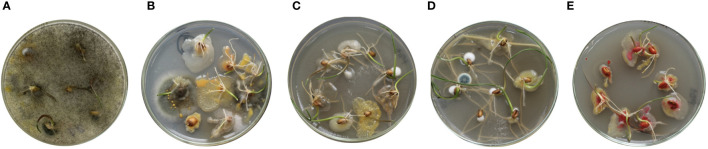
*Triticum aestivum L.* grains with known *Fusarium* spp. infection were pretreated with the test compounds and germinated in a Petri dish. **(A)** Non-treated grains with seed-borne infection. Grains treated with **(B)** 4.5% sodium hypochlorite:water (1:1), **(C)** 0.05% M451, **(D)** 0.1% M451, and **(E)** Maxim XL (0.25% fludioxonil and 0.01% for mefenoxam).

We evaluated the effects of the following seed dressings on wheat grains with known *Fusarium* spp. infection: control (4.5% NaOCl:water [1:1 ratio]), 0.05% and 0.1% M451, and Maxim XL (at a fixed concentration corresponding to 0.25% fludioxonil and 0.01% for mefenoxam) (https://www.syngenta-us.com/seed-treatment/maxim-xl; **Figure 2**). Although all tested compounds inhibited microbial growth, 0.1% M451 exhibited the most effective antimicrobial effect. Maxim XL was less effective than 0.1% M451, and 4.5% NaOCl was least effective as an antifungal agent.

### 3.2 Effect of M451 on the major characteristics of wheat grains and seedlings

**Figure 3:**
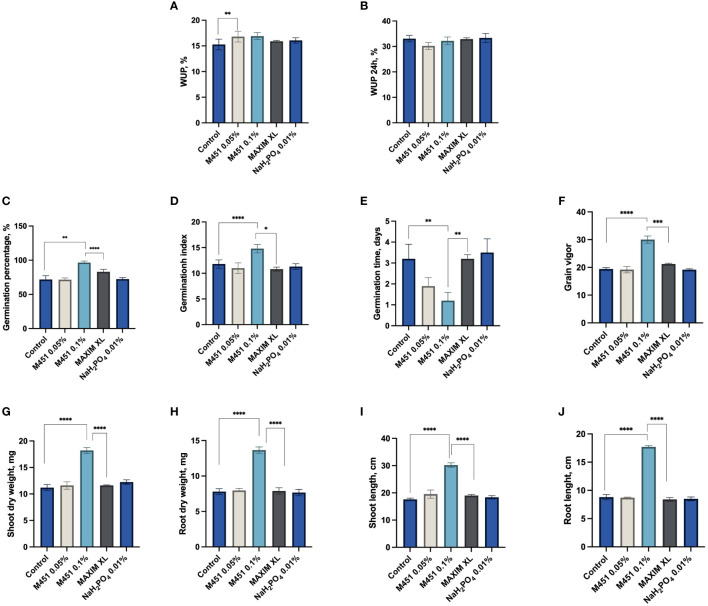
Effects of test compounds on the functional and physical parameters of wheat grains and seedlings. Effects on **(A, B)** water uptake, **(C)** germination percentage, **(D)** mean germination time, **(E)** germination index, **(F)** grain vigor, **(G)** shoot dry weight, **(H)** root dry weight, **(I)** shoot length, and **(J)** root length. *P<0.05, **P<0.01, ***P<0.001, ****P<0.0001.

Next, we examined how seed dressings with the tested compounds affected different features of early plant development (**Figure 3**). Treatment of wheat grains with 0.1% and 0.05% M451 did not inhibit water uptake within the first 24 h (**Figures 3A, B**). Indeed, treatment with either 0.1% or 0.05% M451 significantly increased the GP and GI by 26% and 21%, respectively (*P*<0.01 and *P*<0.001, respectively (**Figures 3C, D**). Treatment with Maxim XL also increased the GP compared with the control (*P*<0.01); however, this improvement was statistically lower than that induced by treatment with 0.1% M451 (*P*<0.05). Treatment with Maxim XL did not affect the GI. Treatment with 0.05% and 0.1% M451 substantially reduced the MGT by 40% and 63%, respectively; however, the difference was statistically significant only for treatment with 0.1% M451 (**Figure 3E**). Similarly, treatment with 0.1% M451 increased the grain vigor by 35% (*P*<0.0001), although this parameter was not changed by the other treatments (**Figure 3F**). Finally, on day 7, treatment with 0.1% M451 significantly increased shoot and root length and dry weight of seedlings by 44–50% (*P*-values ranging from<0.05 to<0.0001) compared with those of the untreated control. These parameters were not affected by treatment with Maxim XL (**Figures 3G–J**).

### 3.3 Effect of M451 on endophytic bacteria and fungi

**Figure 4:**
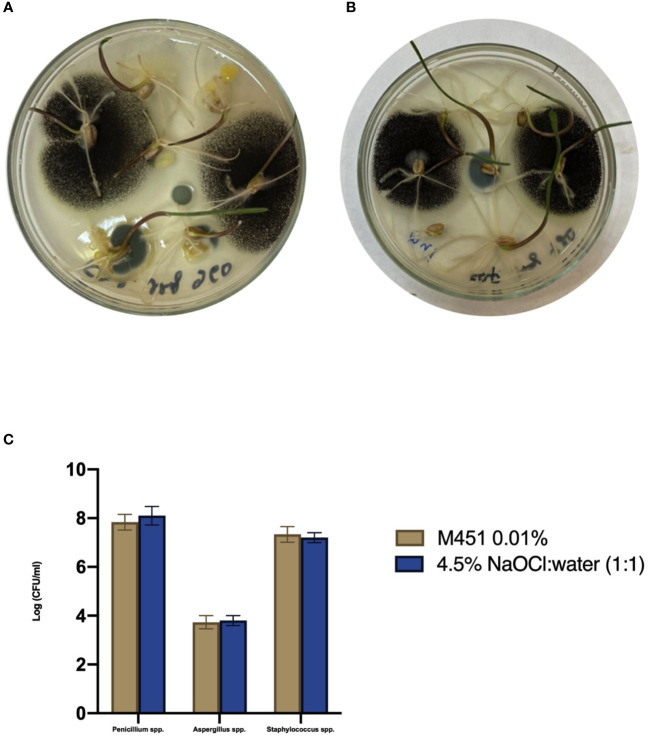
Effect of the M451 seed dressing on the growth of endophytic microorganisms. Wheat grains pretreated with the test compounds were germinated in a Petri dish. **(A)** Grains treated with 4.5% NaOCl:water (1:1). **(B)** Grains treated with 0.1% M451. **(C)** Effects of the test compounds on the growth of endophytes.

Since M451 is a broad-spectrum antimicrobial agent, we examined whether it would negatively affect endophytic bacteria and fungi in wheat seedlings through seed dressing with 0.1% M451 for 30 min. The results were compared with those of grains sterilized with 4.5% NaOCl:water (1:1) for 20 min, which is known not to inhibit endophytic microorganisms (**Figure 4**) (Sahu et al., 2022). MALDI-TOF analysis of the microbial species revealed that neither treatment inhibited the growth of well-known endophytes, such as *Aspergillus* spp., *Penicillium* spp., and *Staphylococcus* spp Toghueo and Boyom, 2020; Patel at al., 2021; Pang at al., 2022.

### 3.4 Lack of penetration and accumulation of M451 in wheat plants

**Figure 5:**
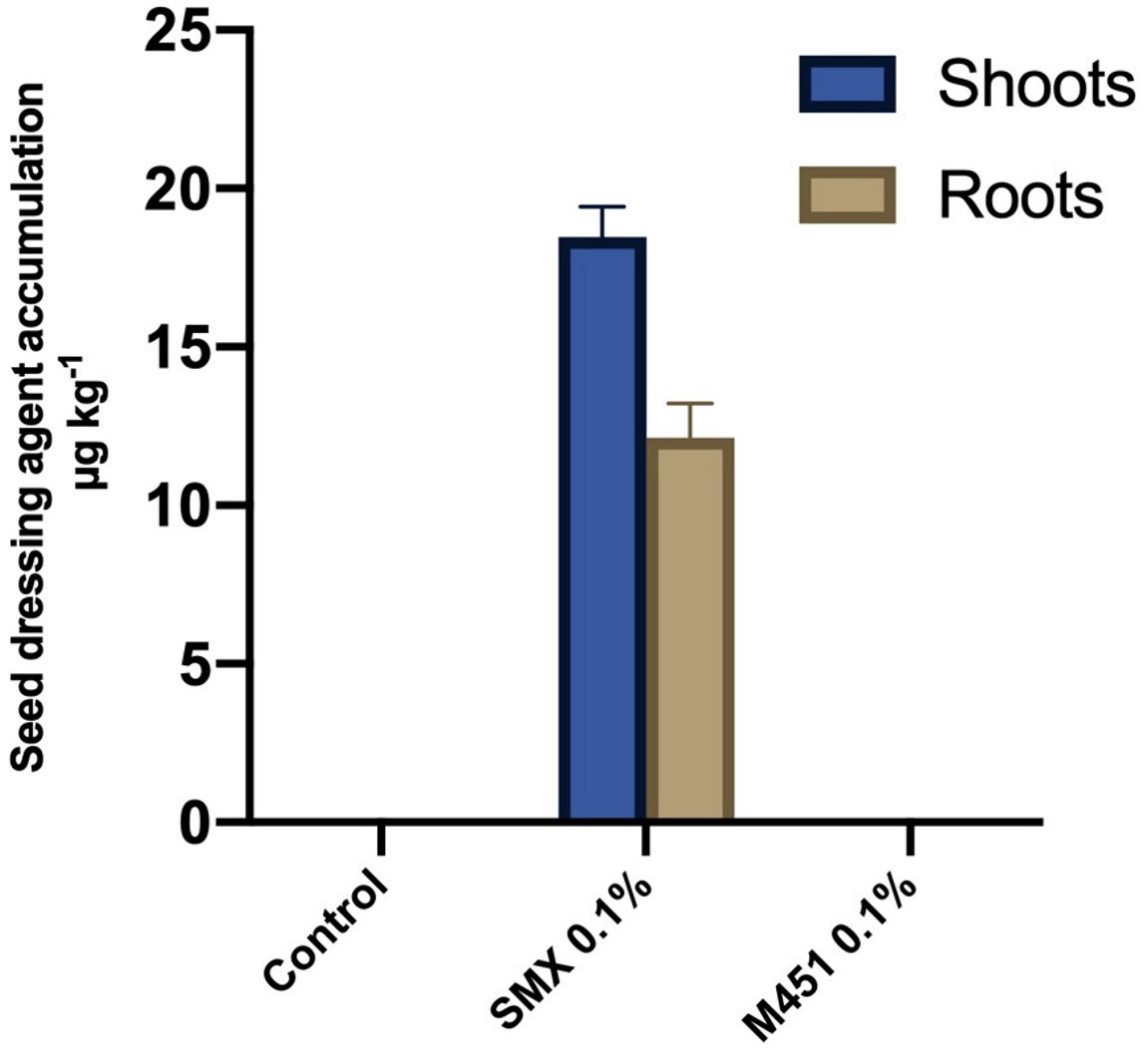
UPLC-MS/MS-based detection of 0.1% M451 and 0.1% sulfamethoxazole (SMX; positive control) in wheat shoots and roots after seed dressing. The data are representative of results from three independent experiments.

For the quantitative analysis of M451 uptake by wheat plants, we analyzed the shoots and roots of wheat seedlings treated with 0.1% M451 or 0.1% sulfamethoxazole (positive control) seed dressings. UPLC-MS/MS analysis indicated that the concentration of sulfamethoxazole in the samples ranged from 10.5 to 20.1 µg·kg^−1^. However, there was no quantifiable uptake of M451 (**Figure 5**).

### 3.5 Effect of M451 on plant characteristics in soil infected with *Fusarium*

**Figure 6:**
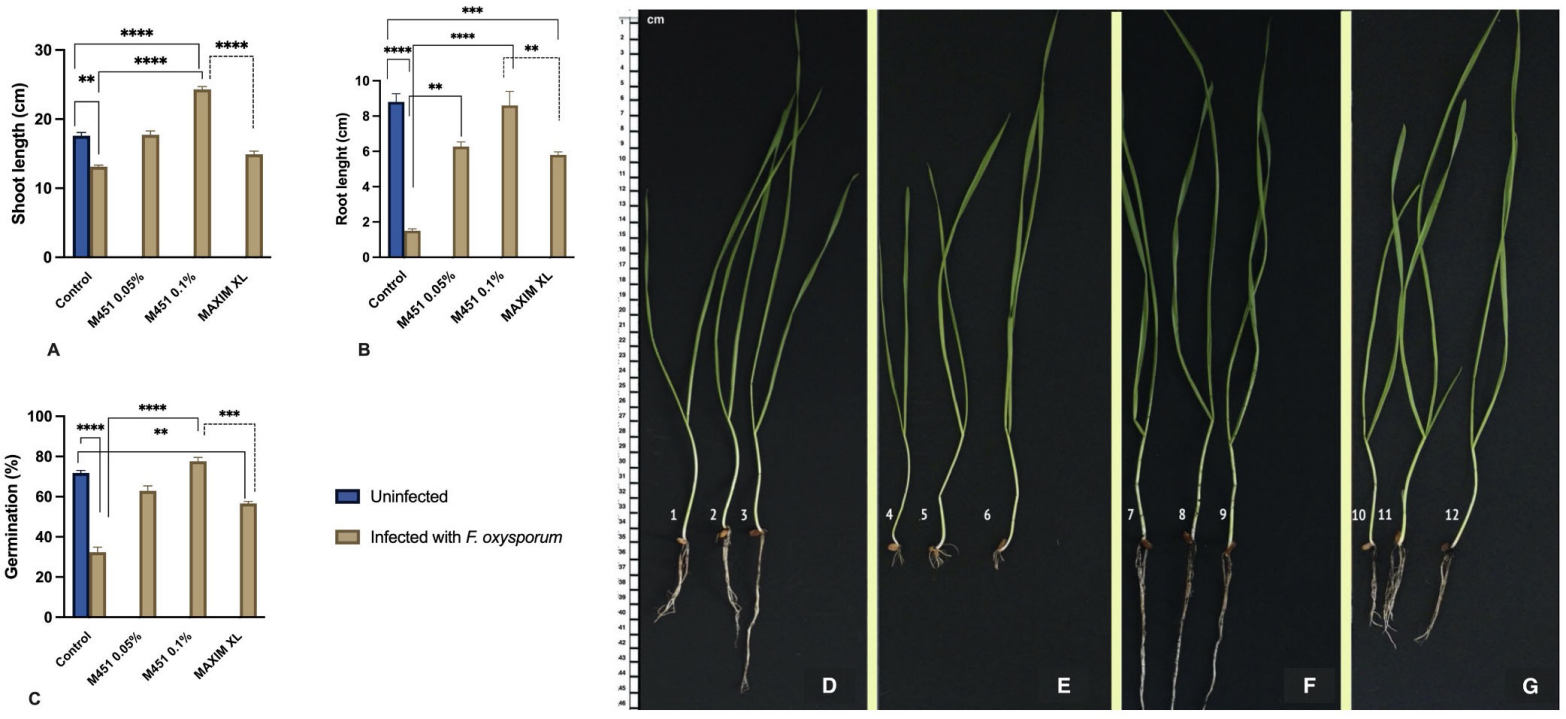
Effect of M451 on plant characteristics in a soil-borne *Fusarium* model of wheat plants. **(A–C)** Effects of M451 and Maxim XL on major characteristics of wheat grains grown in uninfected and *Fusarium*-infected soil. **(A)** Shoot length, **(B)** root length, and **(C)** germination percentage. **(D–G)** Representative images of *Triticum aestivum L.* seedlings germinated from untreated grains or grains treated with M451 and grown in uninfected or *Fusarium*-infected soil. **(D)** Untreated healthy seedling, **(E)** Untreated seedling with soil-borne *Fusarium*, **(F)** healthy seedling treated with 0.1% M451, and **(G)** M451-treated seedling grown in *Fusarium*-infected soil. **P<0.01, ***P<0.001, ****P<0.0001.

Treatment with M451 not only protected the plants from seed-borne infections, but also increased seedling growth in non-infected soil. Therefore, we evaluated the effects of the M451 seed dressing on seedling germination and growth in *Fusarium*-infected soil. Infection with soil-borne *F. oxysporum* reduced the GP of untreated control grains by 54.8% (*P*<0.001) (**Figure 6A**). The negative impact of fungal infection was slightly alleviated when the wheat grains were pretreated with Maxim XL. However, even in this case, the GP of Maxim XL-treated grains was reduced by >21% (not statistically significant) compared with that of uninfected grains. M451 was the most effective agent in this regard. Seed dressing with 0.05% M451 reduced the GP by 12.5%. As discussed in 3.2, seed dressing with 0.1% M451 significantly increased the GP of wheat grains in non-infected soil. Even in *Fusarium*-infected soil, the GP of grains treated with 0.1% M451 was 8% higher (not statistically significant) than that of control grains in non-infected soil.

We also evaluated the effect of soil-borne infection on seedling characteristics. As expected, soil-borne *Fusarium* reduced the shoot and root lengths of seedlings sprouting from untreated grains by 25.5% and 88%, respectively (**Figures 6B, C**). Under the same conditions, pretreatment with Maxim XL only reduced the negative impact of *Fusarium* infection on the shoot and root length, which were reduced by 15.3% and 34%, respectively, compared with the untreated control in uninfected soil. M451 was significantly more effective in alleviating the negative effects of soil-borne *Fusarium* infection on seedling growth. In wheat seedlings that sprouted from grains treated with 0.05% M451, the shoot length was 1% higher and the root length was 24% lower than that of the untreated control in uninfected soil (**Figures 6B, C**). Treatment with 0.1% M451 had the greatest protective effect, inducing an 11% increase in shoot length and only a 1.2% decrease in root length compared with the seedlings of untreated grains in uninfected soil (**Figures 6B, C**). Image analysis of the root structure showed that seed dressing with 0.1% M451 improved the characteristics of the whole root system, including root length, root volume, and root number (**Figures 6D–G**).

### 3.6 Effect of M451 on the biochemical characteristics of wheat seedlings

**Figure 7:**
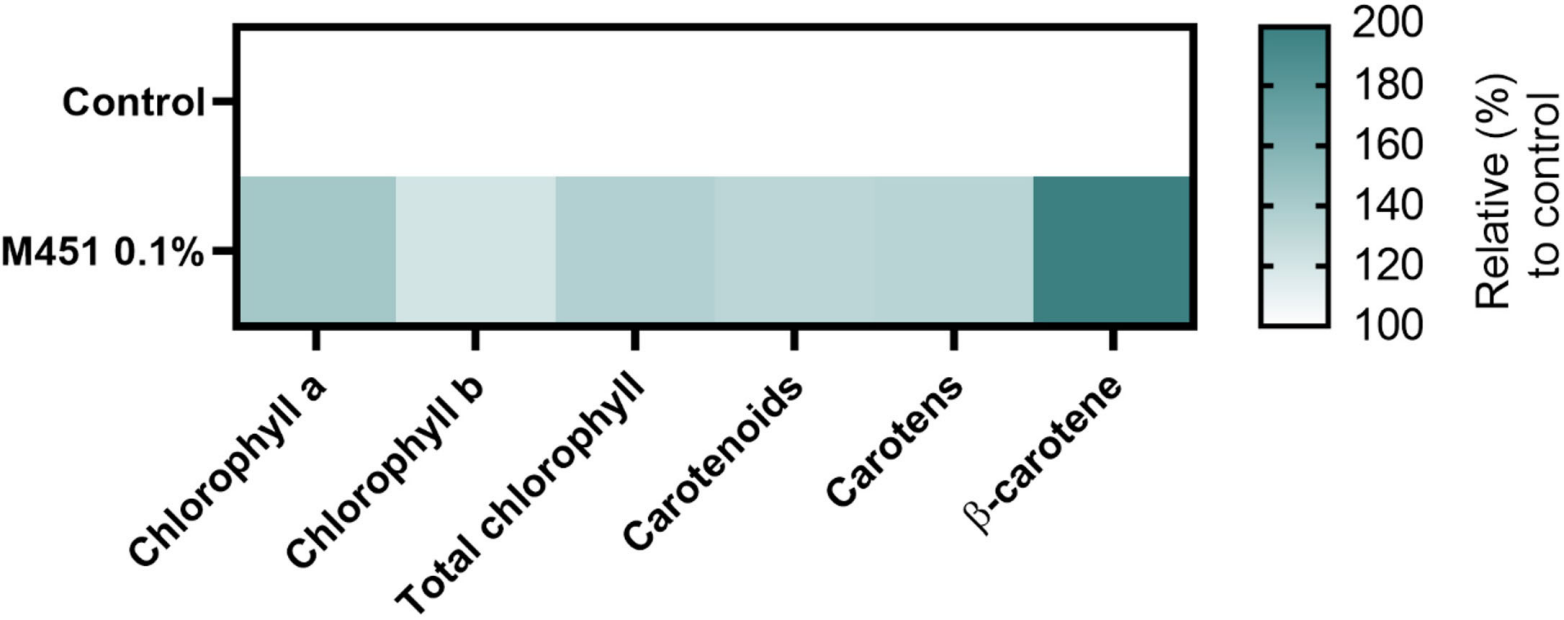
Phytochemical profile of 15-d-old wheat seedlings. The heatmap demonstrates an increase or decrease in the mean value of each parameter (%). The value of each parameter was normalized to the value of the same parameter in the control group. The color scale ranges from white (minimum) to dark green (maximum). The data are representative of results from three independent experiments.

Finally, we examined the effects of M451 treatment on 15-d-old *Triticum aestivum* L. seedlings grown from M451-dressed grains (**Figure 7**). The concentrations of chlorophyll a, chlorophyll b, and total chlorophyll increased in seedlings from M451-treated grains by 42.7%, 20.5%, and 36.1.%, respectively (all *P*<0.01) Moreover, pretreatment of seeds with M451 increased the levels of carotenoids, carotens, and β-carotene by 32.9%, 32.4%, and 95.2%, respectively (all *P*<0.01). These results clearly indicated that treating wheat seeds—even non-infected seeds—with M451 changed their specific phytochemical characteristics.

## 4 Discussion

New fungicides with a broad spectrum of antifungal activity can be used to treat different infections in wheat. There is an urgent need to develop novel broad-spectrum fungicides for agricultural use; however, antifungal drug discovery has always been challenging owing to the difficulties in identifying unique targets for antifungal antibiotics (Tetz and Tetz, 2022; Kitchen et al., 2016; Lugtenberg et al., 2016). In addition, there is an insufficient level of contemporary understanding of affected regulatory pathways, which are shared across pathogenic fungi and other eukaryotic organisms. As a result, today, many antifungals, such as azoles or polyenes that are used in agriculture, are the same as those used in human medicine (Fisher et al., 2022). This represents a particular problem and is a key driver of environmental drug resistance in contaminated soil and crops, resulting in a cross-resistance to medicinal drugs (Jauregi et al., 2021). Previous studies confirmed that the use of environmental azole antifungals triggers the acquisition of the *cyp51A* gene in *Aspergillus* spp., rendering them resistant to medicinal azoles, including voriconazole (Ren et al., 2017). Therefore, the development of agriculture- vs. medicinal-specific fungicides is essential.

M451 is a combination product of a novel antimicrobial component, TGV28, which was specifically developed for agriculture and not being used for the treatment of human diseases, and NaH_2_PO_4_ (0.01%). Tetz et al. (2023) previously reported the inhibitory effects of M451 on multiple plant pathogens. However, this is the first study to evaluate the effects of M451 seed dressing on seedling growth in wheat plants.

We observed an apparent beneficial effect of M451 seed dressing applied to uninfected grains on wheat seedlings, including stimulated germination and the promotion of other critical properties. These included an improved germination percentage, germination index, and mean germination time, as well as a significant increase in shoot and root lengths. Under the same general treatment conditions, none of these properties were improved by treatment with Maxim XL. M451 also did not have any negative effects on water uptake by wheat seedlings, which is known to be a frequent negative effect of fungicides (Biswas and Haynes, 1970; Zhang et al., 2010; Hadas, 1976; Wolny et al., 2018).

We were particularly interested in evaluating the effects of M451 seed dressing on *F. oxysporum* since it is one of the top ten fungal plant pathogens that affects the yield of various plants and possesses a high level of resistance to different antifungals (Doehlemann et al., 2017). Because M451 has been shown to possess broad-range antimicrobial activity, we expected it to be highly protective against seed- and soil-borne *F. oxysporum* infection when used as a seed dressing (Tetz et al., 2023). Consistent with this, wheat seedlings grown from M451-treated grains were significantly less affected by *Fusarium* infection (which partially inhibited the growth of the control seedlings). We also found clear evidence of a dose-dependent effect for M451; treatment with 0.1% M451 conferred a higher level of protection than treatment with 0.05% M451. Under the same conditions, treatment with Maxim XL conferred a significantly lower level of protection against *Fusarium* infection.

We speculate that under conditions of seed- or soil-borne *Fusarium* spp. infection, the seedlings became less sensitive to the fungi, not only because of the direct antifungal activity of M451, but also because M451 enabled the seeds to “outrun” pathogen infection by reducing the time to germination. Indeed, M451 reduced the germination time of uninfected grains, most likely by reducing the duration of dormancy and promoting the transition to the seedling stage. Furthermore, since seedlings are known to have a higher resistance to fungal proliferation, this could lead to a lower sensitivity of the M451 coated grains to *Fusarium* disease.

Another important desirable feature of the agricultural fungicide M451 is that it does not accumulate in plant parts that are consumed by agricultural animals or humans. Various plants, such as tomato, peppers, and rice take up certain fungicides (Matadha et al., 2019; Li et al., 2022; Tao et al., 2022). Here we confirmed that M451 does not penetrate wheat plants and it does not accumulate in wheat seedling tissues. Thus, this means that M451 will not contribute to residual toxicity, making it an attractive antifungal agent for use in edible plants.

Finally, we analyzed the impact of M451 on the microbiome of wheat grains by focusing on the continued presence of fungal endophytes, which are believed to play a crucial role in the habitat adaptability of plants (Ayesha et al., 2021). Despite its broad-spectrum antimicrobial activity, M451 did not inhibit bacterial or fungal endophytes. One potential explanation for this is that because of its high level of activity, M451 is effective even when applied for a short period of time. In this study, we applied M451 to wheat grains for only 30 min. This time period was sufficient for the eradication of pathogenic bacteria and fungi, but was short enough that M451 did not penetrate the seeds deep enough to inhibit the growth of endophytes. These results are particularly interesting considering our previous findings that M451 was effective in eradication of a broad range of agricultural pathogens, including members of the *Ascomycota, Oomycota*, and *Basidiomycota* phyla, within 5 min of exposure (Tetz et al., 2023). This suggests that the 30-min exposure time used in this study could be shortened, further decreasing the potential for nontarget effects of M451 on endophytes. This will be evaluated in follow up studies.

The current study evaluated the potentiation effects of M451 on wheat grain germination and seedling growth under normal and *Fusarium*-infected conditions. Our findings indicate the possibility of developing M451 further into an agriculture-specific antifungal drug candidate through additional *in vivo* research.

## Data availability statement

The original contributions presented in the study are included in the article/**Supplementary Material**. Further inquiries can be directed to the corresponding author.

## Author contributions

VT, KK, and GT conceived and supervised the research. KK and MV conducted experiments. VT, KK, GT, and MV analyzed the data and wrote the manuscript. KK, GT, and VT edited and helped to draft the final manuscript. All authors contributed to the article and approved the submitted version.
