# Supplementary material for: Seed dressing with M451 promotes seedling growth in wheat and reduces root phytopathogenic fungi without affecting endophytes
Source: Front Plant Sci. 2023 May 17;14:1176553. doi: 10.3389/fpls.2023.1176553 (PMC10229829; doi:10.3389/fpls.2023.1176553)
Supplement: Supplementary file 1 [file Table_1.docx]

**Supplementary Table 1.** Chromatographic and mass spectrometric conditions for the analysis of M451

| Mobile phase | (А) MeOH; (B) 0.2% formic acid in H_2_O | | |
| --- | --- | --- | --- |
| Gradient | Isocratic: A, 40%; B, 60% | | |
| Flow rate | 0.4 ml/min | | |
| Injection volume | 10 μl | | |
| Column Temperature | 40 ^о^С | | |
| CI for M451 components | 4–5 min | | |
| Recommended marker ions (MH^+^) | 765.6 | 906.7 | 1047.8 |
|  | 1188.9 | 1330.0 | 1471.1 |
| MRM parameters | MH^+^ - 157 → (M^2^H^+)^ | | |
