# Supplementary material for: Seed dressing with M451 promotes seedling growth in wheat and reduces root phytopathogenic fungi without affecting endophytes
Source: Front Plant Sci. 2023 May 17;14:1176553. doi: 10.3389/fpls.2023.1176553 (PMC10229829; doi:10.3389/fpls.2023.1176553)
Supplement: Supplementary file 2 [file Table_2.docx]

**Supplementary Table 2.** Wavelengths used to measure chlorophyll and carotenoid content

| Measured parameters | Wavelength (nm) |
| --- | --- |
| Chlorophyll a | 645 |
| Chlorophyll b | 662 |
| Carotenoids | 440 |
| Carotenes | 454 |
| β-carotene | 454 |
| Lycopene | 503 |
